# Clinical characteristics, treatment patterns, and outcomes of hospitalized patients with acute heart failure in central Ethiopia: a retrospective observational study

**DOI:** 10.1186/s12872-024-03905-z

**Published:** 2024-05-09

**Authors:** Gashaw Solela, Yimer Seid Yimer

**Affiliations:** 1 Department of Internal Medicine, Yekatit 12 Hospital Medical College, Addis Ababa, Ethiopia; 2 Department of Preventive Medicine, School of Public Health, Addis Ababa University, Addis Ababa, Ethiopia

**Keywords:** Acute heart failure, Clinical characteristics, Treatment patterns, Outcomes, Central Ethiopia

## Abstract

**Background:**

Acute heart failure is the rapid onset of new or worsening symptoms and signs of heart failure. Despite the increasing burden of heart failure in developing countries like Ethiopia, there is a paucity of comprehensive data regarding the clinical characteristics, treatment patterns, and outcomes of acute heart failure, especially in the selected study area. Therefore, this study aimed to assess the clinical characteristics, treatment patterns, and outcomes of hospitalized patients with acute heart failure at Yekatit 12 Hospital Medical College, Addis Ababa, Ethiopia.

**Methods:**

This is a retrospective cross-sectional study of 303 acute heart failure patients who were admitted to the medical wards and intensive care unit of Yekatit 12 Hospital Medical College, Addis Ababa, central Ethiopia, from July 1, 2022, to July 1, 2023. A pretested data abstraction format was used for data extraction from electronic medical records, and SPSS version 26 was used for data analysis. Descriptive analysis was used to summarize sociodemographic data, clinical characteristics, treatment patterns, and outcomes of acute heart failure. Bivariate and multivariate logistic regression models were fitted to identify factors associated with in-hospital mortality. The odds ratio (OR) with the corresponding 95% confidence interval (CI) was calculated to show the strength of the association.

**Results:**

Of the 303 patients, 51.5% were females, and the mean age was 56.7 years. The most frequent symptom and sign were dyspnea (98.7%) and peripheral edema (79%), respectively. The commonest underlying cause and precipitating factor of acute heart failure were cor pulmonale (22.8%) and pneumonia (35.3%), respectively. The commonest anti-remodeling medications prescribed on discharge were beta-blockers (47.9%), followed by mineralocorticoid receptor antagonists (42.8%) and angiotensin-converting enzyme inhibitors/angiotensin receptor blockers (38.6%), and the least prescribed were sodium-glucose cotransporter 2 inhibitors (8.3%). The in-hospital mortality rate was 8.6%, and the median length of hospital stay was 9 days. Based on the multivariate logistic regression analysis, the most important predictors of in-hospital mortality were systolic blood pressure (SBP) < 115 mmHg (adjusted odds ratio [AOR] = 6.28; 95% CI: 1.99, 19.78), chloride level < 96 mg/dL (AOR = 4.88; 95% CI: 1.30, 18.33), blood urea nitrogen (BUN) > 20 mg/dl (AOR = 5.48; 95% CI: 1.47, 20.49), and presence of dyslipidemia (AOR = 3.73, 95% CI: 1.15, 12.07).

**Conclusions:**

This study has shown that systolic blood pressure (SBP) < 115 mmHg, blood urea nitrogen (BUN) > 20 mg/dL, chloride (Cl) level < 96 mg/dL, and the presence of dyslipidemia were statistically significant factors associated with in-hospital mortality among patients with acute heart failure. Hence, healthcare providers should stratify patients with acute heart failure upon admission based on their risk of in-hospital mortality and address those potential negative prognostic indicators accordingly.

## Introduction

According to the European Society of Cardiology guidelines, heart failure (HF) is a clinical syndrome characterized by a combination of symptoms and signs, frequently brought on by a structural and/or functional cardiac abnormality, resulting in decreased cardiac output and/or elevated intracardiac pressures [1]. Acute heart failure (AHF) is characterized by a sudden start of new heart failure (HF) symptoms or worsening of existing ones and by an increase in plasma natriuretic peptide levels [2].

In affluent nations, the prevalence of HF in adult populations ranges from 1 to 3%; however, the exact prevalence in South America and Africa is unknown [3]. There were 56.19 million prevalent cases of HF reported worldwide, according to a secondary analysis of the Global Burden of Disease 2019 research [4]. There are geographic variations in the epidemiology of HF; in 2019, the greatest age-standardized point prevalence of HF (per 100,000 people) was found in high-income North America (1154.09), followed by East Asia (1014.06), Oceania (827.64), and sub-Saharan Africa (482.7 to 812.6). In contrast, the lowest age-standardized rates of prevalence of HF were seen in South Asia (389.97], high-income Asia Pacific (445.28), and Andean Latin America (453.01) [4].

Due to the rising prevalence of cardiovascular diseases and risk factors for HF, such as obesity, dietary habits, and risk factors like hypertension (HTN), ischemic heart disease (IHD), and diabetes mellitus (DM), HF has become an epidemic in Africa [5]. There is a lack of population-based incidence and prevalence studies regarding HF in Sub-Saharan Africa (SSA), but the reported hospital prevalence studies indicate that HF is responsible for 9.4–42.5% of all medical admissions [5].

In the SSA Survey of HF (THESUS–HF) done on 1006 patients who presented with AHF and were admitted to 12 university hospitals in 9 countries, including Ethiopia, the commonest causes of HF were hypertensive heart disease (HHD) and rheumatic heart disease (RHD), accounting for 45.4% and 14.3% of all causes of AHF, respectively. Common comorbidities identified in this registry were atrial fibrillation (18.3%), anemia (15.2%), DM (11.4%), and renal dysfunction (7.7%) [6]. According to the prospective, multicenter INTERnational Congestive Heart Failure Study (INTER-CHF), which included a total of 1,294 patients with HF from Nigeria (383 patients), South Africa (169 patients), Sudan (501 patients), Uganda (151 patients), and Mozambique (90 patients), the most frequent causes of HF were IHD and HHD, which were seen in 20% and 35% of the patients, respectively [7].

Studies in Ethiopia have reported inconsistent findings regarding the underlying causes, treatment patterns, and outcomes of AHF. A study from the University of Gondar Comprehensive Specialized Hospital (UGCSH), showed that IHD was the leading cause of AHF, seen in 27% of the patients [8], whereas studies done at St. Paul’s Hospital Millennium Medical College (SPHMMC) and Tikur Anbessa Specialized Hospital (TASH), showed that RHD was the leading cause of HF, seen in 30% and 48.5% of the patients, respectively [9, 10]. The commonest anti-remodeling agents prescribed on discharge were spironolactone and angiotensin-converting enzyme (ACE) inhibitors in the studies from UGCSH and SPHMMC [8, 9], whereas spironolactone and beta-blockers (BBs) were the commonest anti-remodeling agents prescribed in a study from Dessie Referral Hospital (DRH) [11]. High in-hospital mortality rates of AHF were observed in studies from SPHMMC (24.4%) [9] and DRH (22.9%) [11], followed by Jimma University Medical Centre (21.3%) [12], TASH (17.2%) [10], and UGCSH (10.6%) [8].

Previous studies conducted in Ethiopia were incomprehensive and showed inconsistent reports regarding the clinical profiles, underlying causes, comorbid conditions, treatment patterns, and in-hospital mortality of AHF, demanding more studies to narrow the heterogeneity of these findings. Therefore, this study aimed to assess the clinical characteristics, treatment patterns, and outcomes of hospitalized patients with AHF at Yekatit 12 Hospital Medical College, Addis Ababa, Central Ethiopia. The findings will be helpful for a better and more comprehensive understanding of the clinical characteristics, treatment patterns, and outcomes of AHF in Ethiopia.

## Methods

### Study design, area and period

A retrospective cross-sectional study was conducted on patients with acute heart failure (AHF) who were admitted to the medical wards and intensive care unit (ICU) of Yekatit 12 Hospital Medical College (Y12HMC), Addis Ababa, central Ethiopia, from July 1, 2022, to July 1, 2023. Y12HMC is one of the biggest public referral hospitals in Addis Ababa, with a total of 456 beds. It delivers both clinical and academic services in more than six specialties. The hospital has four major departments, namely internal medicine, surgery, pediatrics, gynecology, and obstetrics. The internal medicine department has a medical emergency unit, three general medical wards, an isolation ward, and a medical ICU. Patients indicated for hospitalization due to AHF are directly admitted to the medical wards or medical ICU and are managed by a team of nurses, interns, general practitioners, residents, internists, and cardiologists.

### Study population

All patients who were ≥ 15 years of age, diagnosed with AHF, and admitted to the medical wards and ICU of Y12HMC during the study period.

### Eligibility criteria

Eligible patients were those aged ≥ 15 years old, diagnosed with AHF, and admitted to the medical wards and ICU of Y12HMC during the study period. Patients without documentation of admission or discharge notes and those who were discharged within 24 hours were excluded since such patients were expected to have incomplete records on their clinical, investigation, and outcome characteristics.

### Sample size and sampling method

Sample size was calculated using a single population proportion sample size determination formula, assuming a mortality rate of 24% from a previous study [8], a 95% confidence level (CI), and a 5% margin of error (d).

$${\rm{n = }}\frac{{{{\rm{Z}}_{{\rm{\alpha /2}}}}^{\rm{2}}{\rm{ \times p }}\left( {{\rm{1 - p}}} \right)}}{{{{\rm{d}}^{\rm{2}}}}}{\rm{ = }}\frac{{{\rm{1}}{\rm{.9}}{{\rm{6}}^{\rm{2}}}{\rm{ \times 0}}{\rm{.24 }}\left( {{\rm{1 - 0}}{\rm{.24}}} \right)}}{{{{\left( {{\rm{0}}{\rm{.05}}} \right)}^{\rm{2}}}}}{\rm{ = 280}}$$

After adding 10% for the probability of having incomplete data, the sample size was found to be 308. A convenient sampling method was used to extract all the available medical records of the eligible patients from July 1, 2022, to July 1, 2023.

### Study variables

The dependent variable was in-hospital mortality (yes or no) due to AHF.

The independent variables were:

#### Socio-demographic data and medical history

Age, sex, marital status, tobacco consumption, alcohol consumption, body mass index, prior hospitalization for HF, and duration of HF.

#### Precipitating factors

Pneumonia, drug discontinuation, urinary tract infection (UTI), acute myocardial infarction (MI), infective endocarditis, atrial fibrillation, thyrotoxicosis, anemia, and uncontrolled HTN.

#### Comorbid conditions

HTN, atrial fibrillation, stroke, IHD, DM, chronic kidney disease (CKD), chronic obstructive pulmonary disease (COPD)/asthma, post TB lung disease, post coronavirus disease 2019 (COVID-19) lung fibrosis, chronic pulmonary thromboembolism (PTE), human immunodeficiency virus/acquired immunodeficiency syndrome (HIV/AIDS), thyroid disorder, dyslipidemia, obesity, hyperuricemia/gout, anemia, and cancer.

#### Clinical characteristics

New York Heart Association (NYHA) functional class on admission, dyspnea, orthopnea, paroxysmal nocturnal dyspnea, systolic blood pressure (SBP), diastolic blood pressure (DBP), pulse rate, respiratory rate, oxygen saturation, raised jugular venous pressure or neck vein distension, rales/crepitations, S3 gallop, and peripheral edema.

#### Laboratory data

Sodium (Na), potassium (K), chloride (Cl), hemoglobin, blood urea nitrogen (BUN), serum creatinine, estimated glomerular filtration rate (eGFR), random blood sugar (RBS), glycated hemoglobin (HbA1c) and thyroid stimulating hormone (TSH).

#### Imaging data

Chest x-ray [CXR] (signs of heart failure or pneumonia), electrocardiogram (atrial fibrillation/QTc interval/QRS duration) and echocardiographic (left ventricular ejection fraction [LVF], diastolic dysfunction, pulmonary hypertension [PH], right ventricular dysfunction, valvular lesions, pericardial thickening /effusion) data.

### Data collection procedures

Data were extracted from the electronic medical record system by trained general practitioners using a pretested data abstraction format that was prepared by reviewing similar studies, and the data collection process was closely monitored by the principal investigator. Sociodemographic variables, medical history, causes, precipitating factors, comorbid conditions, clinical presentations, laboratory tests, imaging (chest X-ray, electrocardiogram, echocardiography) studies on admission, medications used before presentation, during hospitalization, and on discharge, and treatment outcomes were all extracted from electronic medical records and documented in the data abstraction format.

### Data quality control technique

The quality of the data was ensured through the training of data collectors, close supervision, and prompt feedback. To assure the completeness of the data abstraction format, a pre-test was conducted among 5% (15) of the patients in the study area, and the proper modifications were made to the format, where variables with no available data like educational status and income were removed. The data were checked for inconsistencies, coding errors, completeness, accuracy, clarity, and missing values, and appropriate corrections were made by the principal investigator.

### Operational definitions

Acute heart failure (AHF): a clinical syndrome characterized by a sudden start of new heart failure symptoms or worsening of the existing ones [2].

New-onset HF: Development of HF symptoms and signs in patients without a history of HF [2]. Acute decompensated heart failure (ADHF): Worsening of HF symptoms and signs in patients with a prior history of HF [2].

Outcomes of AHF: Dead, improved, referred, absconded or left against medical advice.

In-hospital mortality: Death of a patient with acute HF due to any cause, occurring after admission to the medical ward or ICU.

The NYHA functional classification: Categorizes HF on a scale of I to IV; Class I: no limitation of physical activity, Class II: slight limitation of physical activity, Class III: marked limitation of physical activity, and Class IV: occurrence of symptoms even at rest [13].

Ejection fraction (EF) categories: HF with reduced EF (HFrEF) when EF is less than or equal to 40%, HF with mildly reduced EF (HFmrEF) when EF ranges between 41% and 49% and HF with preserved EF (HFpEF), when EF is greater than or equal to 50% [13].

### Data entry and statistical analysis

Statistical Program for Social Sciences (SPSS) version 26 was used to enter and analyze the data. A descriptive analysis was performed to describe the clinical characteristics, treatment patterns, and in-hospital outcomes of patients. Continuous variables were expressed as mean (SD) when normally distributed or median (IQR) when not normally distributed. Categorical variables were also presented as frequency and percentage. Bivariate and multivariate logistic regression models were fitted to identify the contributing factors to in-hospital mortality secondary to AHF. The multivariate logistic regression model assumptions were fulfilled, as verified by a non-significant Hosmer and Lemeshow test. Multiple imputation was used to manage the missing data. To demonstrate the strength of the association between predictor and outcome variables, the crude odds ratio (COR) and adjusted odds ratio (AOR), together with the 95% confidence interval (CI), were computed. Predictors with a *P* value < 0.25 in the bivariate analysis were subsequently considered in the multivariate model. A *P*-value less than 0.05 was regarded as statistically significant.

## Results

### Socio-demographic characteristics and medical history

There were a total of 2351 annual medical admissions to the medical wards and medical ICU of Yekatit 12 Hospital Medical College in a year from July 1, 2022, to July 1, 2023. Of these admissions, 311 (13.2%) were due to acute heart failure and 303 AHF patients were found to be eligible and included in this study.

Of the 303 AHF patients, 92.7% were admitted to the medical wards, and 7.3% were admitted to the medical ICU. The mean age of the patients was 56.7 years (SD: 18.4), which was higher in patients with LVEF > 40% than those with LVEF ≤ 40%; the minimum age was 15 years and the maximum age was 100 years; and nearly half (51.5%) were females. Elderly patients (≥ 65 years old) comprised 37% of all AHF patients. The median body mass index (BMI) was 23 kg/m^2^ (IQR: 21.4–24.8), and 7.9% of the patients were obese (BMI of ≥ 30 kg/m^2^). The duration of HF was less than a year in the majority (64.2%) of patients, and 36% of the patients had a prior history of hospitalization for HF [Table 1].

**Table 1:** Socio-demographic characteristics and medical history of patients with acute heart failure stratified by LVEF at Y12HMC (July 1, 2022 - July 1, 2023)

	Overall no. /total no. (%)	LVEF ≤ 40% no. /total no. (%)	LVEF > 40% no. /total no. (%)	*P* value
AHF annual admission	311/2351 (13.2)			
Venue of care for AHF				0.779
Medical Ward	281/303 (92.7)	79/85 (92.9)	153/163 (93.9)	
Medical ICU	22/303 (7.3)	6/85 (7.1)	10/163 (6.1)	
Female sex	156/303 (51.5)	37/85 (43.5)	91/163 (55.8)	0.066
Mean age (SD) – years	56.7 (18.4)	52.3 (16.3)	59.5 (19.3)	0.004
Age categories (years)				0.029
<20	6/303 (2.0)	3/85 (3.5)	2/163 (1.2)	
20–39	61/303 (20.1)	23/85 (27.1)	28/163 (17.2)	
40–64	122/303 (40.3)	35/85 (41.2)	58/163 (35.6)	
≥65	114/303 (37.6)	24/85 (28.2)	75/163 (46)	
Marital status				0.190
Married	192/266 (63.4)	61/82 (74.4)	97/138 (70.30	
Divorced/separated	14/266 (4.6)	6/82 (7.3)	7/138 (5.1)	
Widowed	9/266 (3.0)	0/82	7/138 (5.1)	
Single	51/266 (16.8)	15/82 (18.3)	27/138 (19.6)	
Median BMI (IQR) – kg/m^2^	23 (21.4–24.8)			0.656
BMI categories				0.097
<18.5	19/299 (6.3)	7/84 (8.3)	11/162 (6.8)	
18.5–24.9	209/299 (69.0)	64/84 (76.2)	103/162 (63.6)	
25-29.9	47/299 (15.5)	10/84 (11.9)	32/162 (19.8)	
≥30	24/299 (7.9)	3/84 (3.6)	16/162 (9.9)	
Tobacco consumption				0.032
Yes	50/303 (16.5)	20/85 (23.5)	21/163 (12.9)	
No	253 (83.5)	65/85 (76.5)	142/163 (87.1)	
Alcohol consumption				< 0.001
Yes	46/303 (15.2)	25/85 (29.4)	11/163 (6.7)	
No	257/303 (84.8)	60 (70.6)	152/163 (93.3)	
Prior hospitalization for HF*				0.070
Yes	110/303 (36.3)	25/85 (29.4)	67/163 (41.1)	
No	193/303 (63.7)	60/85 (70.6)	96/163 (58.9)	
Duration of HF (years)				0.874
0–1	194/302 (64.2)	53/85 (62.4)	101/163 (62)	
>1–2	21/302 (7.0)	5/85 (5.9)	13/163 (8)	
>2–5	48/302 (15.9)	15/85 (17.6)	24/163 (14.7)	
>5	39/302 (12.9)	12/85 (14.1)	25/163 (15.3)	

The *P* values were obtained using the χ^2^-test, independent samples t-test and median test. LVEF, left ventricular ejection fraction; AHF, acute heart failure; ICU, intensive care unit; BMI, body mass index, SD, standard deviation; IQR, interquartile range. *Admission within the previous 2 years.

### Clinical characteristics

Nearly 58% of the patients were diagnosed with ADHF, and the rest were diagnosed with de novo (new-onset) HF. The majority of patients (62.4%) had biventricular HF, and the proportion of patients with left ventricular HF was higher in the group with LVEF ≤ 40% compared to that with LVEF > 40%. Nearly two-thirds of the patients (70.3%) had NYHA class IV HF, and the proportion of patients having class IV HF was higher in the reduced LVEF (≤ 40%) group as compared with that of LVEF > 40%. The commonest symptoms were dyspnea (98.7%) and orthopnea (70%), whereas the commonest signs were peripheral edema (79%), and rales or crepitations (57.4%). The mean SBP was 121 mmHg, and the mean DBP was 74 mmHg, both of which were higher in patients with reduced LVEF as compared with those with LVEF > 40%. Only 2.7% had S3 gallop, which was more common in those patients with reduced LVEF, and only 3% had had cardiogenic shock at the time of presentation [Table 2].

**Table 2:** Clinical characteristics of patients with acute heart failure stratified by LVEF at Y12HMC (July 1, 2022 - July 1, 2023)

Characteristics	Overall (*N* = 303) n (%)	LVEF ≤ 40% (*N* = 85) n (%)	LVEF > 40% (*N* = 163) n (%)	*P* value
Category of acute HF				0.652
New onset HF	128 (42.2)	38 (44.7)	68 (41.7%)	
ADHF	175 (57.8)	47 (55.3)	95 (58.3)	
Type of ventricular HF				< 0.001
Left ventricular HF	44 (14.5)	17 (20.0)	16 (9.8)	
Right ventricular HF	70 (23.1)	2 (2.4)	57 (35.0)	
Biventricular HF	189 (62.4)	66 (77.6)	90 (55.2)	
Cardiogenic shock	9 (3.0)	3 (3.5)	4 (2.5)	0.627
NYHA classification				0.008
II/III	86 (28.4)	15 (17.6)	55 (33.7)	
IV	213 (70.3)	70 (82.4)	108 (66.3)	
Dyspnea	299 (98.7)	84 (98.8)	162 (99.4%)	0.638
Orthopnea	212 (70.0)	62 (72.9)	117 (71.8)	0.846
Paroxysmal nocturnal dyspnea	158 (52.1)	50 (58.8)	82 (50.3)	0.202
Pulse rate > 100(beats/min.)	71 (23.4%)	21 (24.7)	30 (18.4)	0.224
Respiratory rate > 25 (breaths/min.)	48 (15.8)	75 (88.2)	132 (81)	0.144
SO2 < 90% (with room air)	256 (84.5)	70 (82.4)	138 (83.9)	0.639
Systolic BP (mmHg) – mean (SD)	121(22)	122.7 (22.5)	119.6 (21.6)	0.284
Category of SBP (mmHg)				0.148
<115	137 (45.2)	33 (38.8)	79 (48.5)	
≥115	166 (54.8)	52 (61.2)	84 (51.5)	
Diastolic BP (mmHg) – mean (SD)	75 (14)	78.9 (15.3)	72.8 (13.5)	0.001
Category of DBP (mmHg)				0.113
<60	27 (8.9)	5 (5.9)	20 (12.3%)	
≥60	276 (91.1)	80 (94.1)	143 (87.7)	
Raised JVP/jugular vein distension	71 (23.4)	23 (27.1)	42 (25.8)	0.826
Rales/crepitations	174 (57.4)	53 (62.4)	90 (55.2)	0.280
S3 gallop	7 (2.7)	4 (4.7)	1 (0.6)	0.030
Peripheral edema	239 (78.9)	62 (72.9)	135 (82.8)	0.068

The *P* values compare clinical characteristics of acute HF between EF ≤ 40 (*N* = 85) and EF > 40% (*N* = 163), using the χ2-test (categorical variables) and independent samples t-test (continuous variables). Y12HMC, Yekatit 12 Hospital Medical College; LVEF, left ventricular ejection fraction; HF, heart failure; ADHF, acute decompensated heart failure; SBP, systolic blood pressure; DBP, diastolic blood pressure

### Underlying causes, precipitating factors, and comorbid conditions

**Fig. 1:**
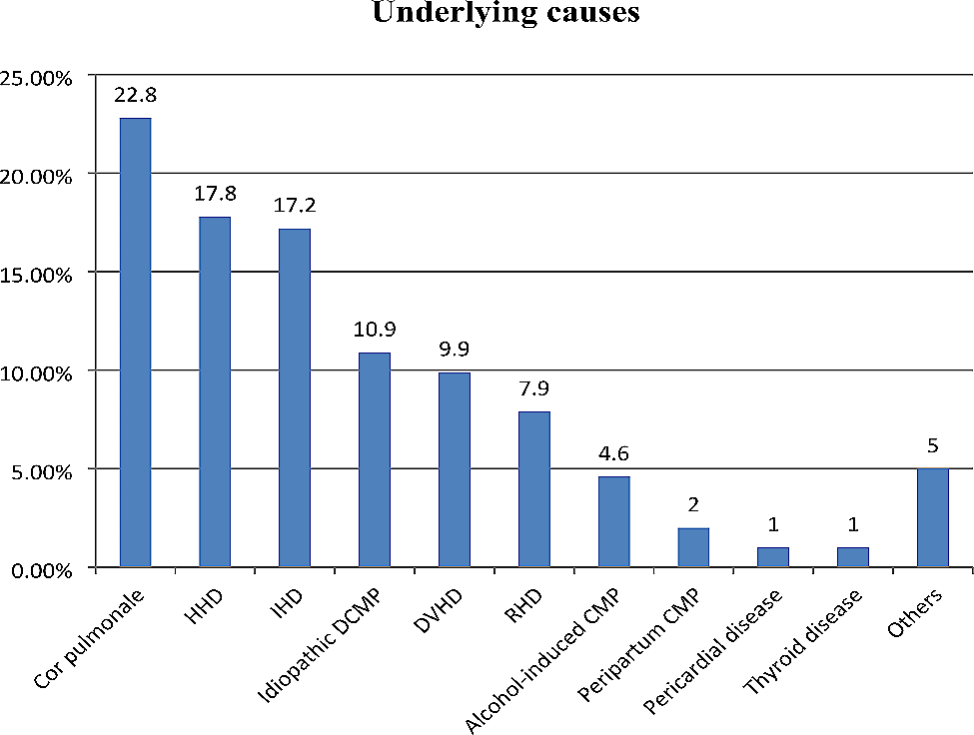
Underlying causes of acute heart failure for hospitalized patients at Y12HMC (July 1, 2022 - July 1, 2023) HHD, hypertensive heart disease; IHD, ischemic heart disease; CMP, cardiomyopathy; DCMP, dilated cardiomyopathy; RHD, rheumatic heart disease. Others include severe anemia, tachycardia-induced CMP, congenital heart disease, and hypertrophic CMP.

The most common underlying cause of AHF was cor pulmonale (22.8%), followed by HHD (17.8%), IHD (17.2%), and idiopathic dilated CMP (10.9%). The other less common causes were degenerative valvular heart disease (9.9%), RHD (7.9%), alcohol-induced CMP (4.6%), peripartum CMP (2%), pericardial disease (1%), and thyroid disease (1%) [Fig. 1].

**Fig. 2:**
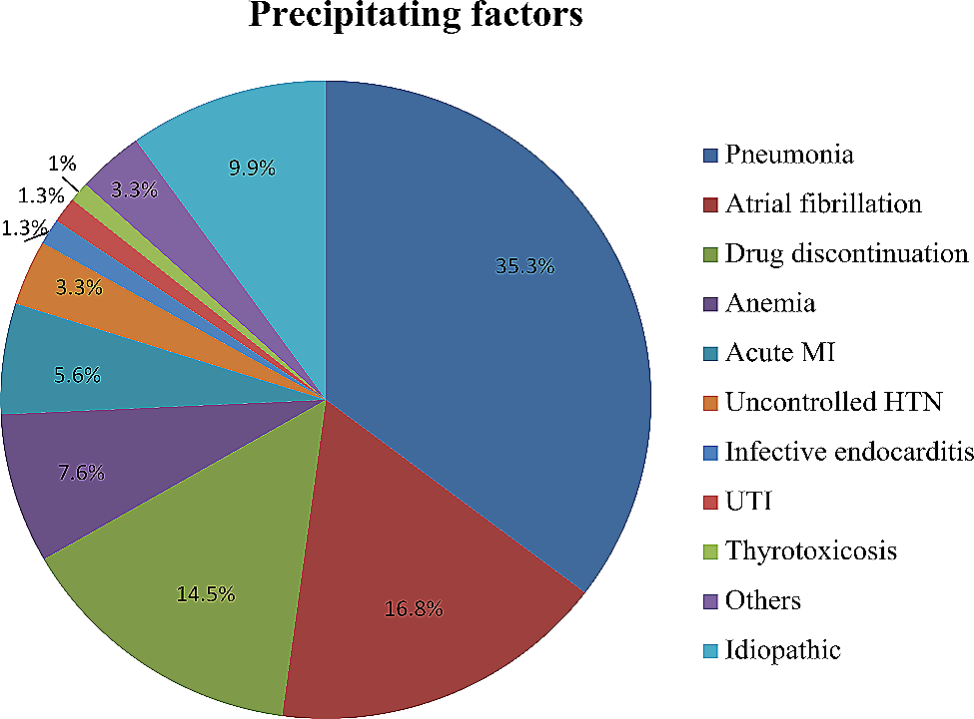
Precipitating factors of acute heart failure for hospitalized patients at Y12HMC (July 1, 2022 - July 1, 2023) MI, myocardial infarction; HTN, hypertension; UTI, urinary tract infection. Others include sub-optimal diuresis, dietary non-adherence, acute gastroenteritis, tuberculosis, pregnancy, and natural progression of the disease

Pneumonia (35.3%) was the most common precipitating factor in AHF, followed by atrial fibrillation (16.8%), drug discontinuation (14.5%), and anemia (7.6%). Other less common precipitating factors were acute MI (5.6%), uncontrolled HTN (3.3%), infective endocarditis (1.3%), UTI (1.3%), and thyrotoxicosis (1%). Of note, precipitating factors were idiopathic or unknown in 9.9% of the patients [Fig. 2].

The major cardiovascular comorbidities of AHF were HTN (48.2%), atrial fibrillation (23.8%), IHD (19.8%), and stroke (3.6%). The major non-cardiovascular comorbidities were DM (21.1%), anemia (17.8%), COPD/asthma (14.9%), and dyslipidemia (13.9%). Less common non-cardiovascular comorbidities were CKD (9.6%), obesity (7.9%), HIV/AIDS (7.3%), post-TB lung disease (6.6%), chronic PTE (6.3%), thyroid disorder (4.6%), hyperuricemia/gout (4.6%), cancer (3.3%), and post-COVID-19 fibrosis (2.6%). A small number of AHF patients (5.6%) had other comorbidities, including tuberculosis, malnutrition, obstructive sleep apnea, interstitial lung disease, chronic liver disease, osteoarthritis, and rheumatoid arthritis.

### Laboratory and imaging data

The commonest electrolyte disorder was hypochloremia, or Cl level < 96 mmol/L (36.6%), followed by hyponatremia, or Na level < 135 mmol/L (35.7%), and hypokalemia, or K level < 3.5 (16.3%). Nearly half (50.6%) of 168 patients, for whom BUN was determined, had raised BUN level (> 20 mg/dL); among 286 patients, for whom Cr was determined, 28% had raised Cr level (> 1.2 mg/dL). Among 280 patients for whom eGFR was calculated, 25% had impaired renal function (eGFR < 60 mL/min/1.73 m2). Among 260 patients for whom aspartate transaminase [AST] was determined, 27.3% had raised AST (> 40 U/L), and of 259 patients for whom alanine transaminase (ALT) was determined, 24.7% had raised ALT (> 40 U/L). Among 300 patients, for whom Hgb levels were available, 28% had Hgb < 12 g/dL. Of the 251 patients for whom RBS was determined, 13.5% had RBS ≥ 200 mg/dL. HbA1c level was done only for 48 patients, and 41.7% of them had HbA1c ≥ 6.5%. TSH level was available only for 26 patients; 15.4% of them had a low TSH level (< 0.05 mIU/L), and 19.2% had a high TSH level (> 7.5 mIU/L) [Table 3].

CXR data was available for 231 patients, and the most commonly identified radiologic findings were cardiomegaly (65.4%) and pleural effusion (61%), followed by pulmonary edema (32.9%) and pneumonia (24.2%). ECG was done for 228 patients, and the most common findings were ST-T wave abnormalities (63.7%), prolonged QTc interval (31.6%), atrial fibrillation (26.9%), and sinus tachycardia (20.3%). Of 248 patients, for whom data on LVEF was available, the majority (60.9%) had preserved LVEF (≥ 50%), 34.3% had reduced LVEF (≤ 40%) and 4.8% had mildly reduced LVEF (41–49%). Of 246 patients, for whom data on pulmonary hypertension (PH) is available, more than half (54.5%) had moderate or severe PH [Table 3].

**Table 3:** Laboratory and imaging data of patients with acute heart failure at Y12HMC (July 1, 2022 - July 1, 2023)

Lab. data – no. /total no. (%)		CXR findings – no. /total no. (%)	
Sodium		Cardiomegaly	151/231 (65.4)
< 135 (mmol/L)	101/283 (35.7)	Pulmonary edema	76/231 (32.9)
> 145	18/283 (6.4)	Pleural effusion	141/231 (61.0)
135–145	164/283 (58)	Pneumonia	56/231 (24.2)
Potassium (mmol/L)		**ECG findings – no. /total no. (%)**	
<3.5	46/282 (16.3)	Sinus tachycardia	46/227 (20.3)
>5	40/282 (14.2)	Atrial fibrillation	62/227 (26.9)
3.5–5	196/282 (69.5)	ST-T wave abnormalities	144/226 (63.7)
Chloride (mmol/L)		QTc interval (ms) – mean (SD)	463 (34.0)
<96	98/268 (36.6)	Prolonged QTc interval	71/225 (31.6)
>106	24/268 (9)	QRS duration (ms) – mean (SD)	95 (23.0)
96–106	146/246 (54.5)	Prolonged QRS duration	35/228 (15.6)
BUN > 20 mg/dL	85/168 (50.6)	Pathologic Q waves	19/228 (8.4)
Creatinine > 1.2 (mg/dL)	80/286 (28.0)	**Echo findings – no. /total no. (%)**	
eGFR (mL/min/1.73m^2^) < 60	70/280 (25.0)	Left ventricular EF (%)	
AST > 40 (U/L)	71/260 (27.3)	≤ 40%	85/248 (34.3)
ALT > 40 (U/L)	64/259 (24.7)	41–49	12/248 (4.8)
Total bilirubin > 1.2 (mg/dL)	38/139 (27.3)	≥50	151/248 (60.9)
Hemoglobin < 12 (g/dL)	84/300 (28.0)	Grade II/III diastolic dysfunction	55/238 (23.1)
TSH (mIU/L)		Right ventricular dysfunction	66/239 (27.6)
<0.05	4/26 (15.4)	Moderate/severe LVH	34/239 (14.2)
>7.5	5/26 (19.2)	Moderate/severe PH	137/246 (54.5)
RBS (mg/dL)		Moderate/severe valve lesions	134/246 (54.3)
140–199	64/251 (25.5)	Pericardial thickening or effusion	17/240 (7.1)
≥200	34/251 (13.5)		
HbA1c ≥ 6.5 (%)	20/48 (41.7)		

BUN, blood urea nitrogen; eGFR, estimated glomerular filtration rate; AST, aspartate aminotransferase; ALT, alanine transaminase; TSH, thyroid stimulating hormone; RBS, random blood sugar; HbA1C, glycated hemoglobin; EF; ejection fraction; ECG, electrocardiography; Echo, echocardiography; LVH, left ventricular hypertrophy; PH, pulmonary hypertension

### Treatment patterns

**Fig. 3:**
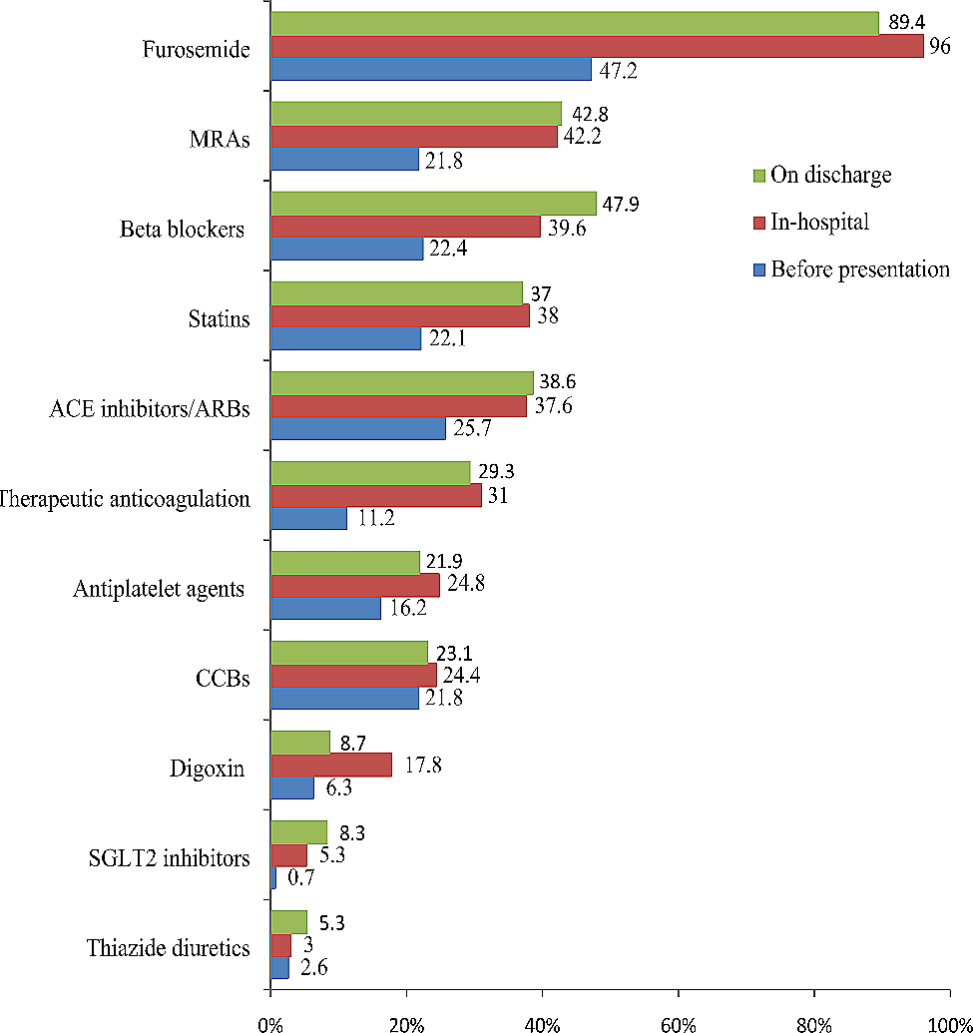
Prescription patterns before presentation, during hospitalization, and on discharge for patients with acute heart failure at Y12HMC (July 1, 2022 - July 1, 2023) MRAs, mineralocorticoid receptor antagonists; ACE, angiotensin-converting enzyme; ARBs, angiotensin receptor blockers; SGLT2, sodium-glucose cotransporter 2; CCBs, calcium channel blockers

The most commonly prescribed medications before presentation were furosemide (47.2%) and ACE inhibitors/angiotensin receptor blockers [ARBs] (25.7%), followed by beta blockers [BBs] (22.4%), statins (22.1%), mineralocorticoid receptor antagonists [MRAs] (21.8%), and calcium channel blockers [CCBs] (21.8%). The most commonly prescribed medications during hospitalization were furosemide (96%) and MRAs (42.2%), followed by BBs (39.6%), statins (38%), and ACE inhibitors/ARBs (37.6%) [Fig. 3]. Additional therapies given during hospitalization were prophylactic anticoagulation (49.2%), vasopressor or inotropic support (9.2%), and ventilator support (2%). Of note, more than half of the patients (62%) were prescribed antibiotics in the course of their hospital stay.

Among the anti-remodeling agents prescribed on discharge, the most prescribed were BBs (47.9%), followed by MRAs (42.8%) and ACE inhibitors/ARBs (38.6%), and the least prescribed were SGLT2 inhibitors (8.3%). Prescription of BBs was much better on discharge (47.9%) compared to before presentation (22.4%) and during hospital stays (39.6%). [Figure 3].

**Fig. 4:**
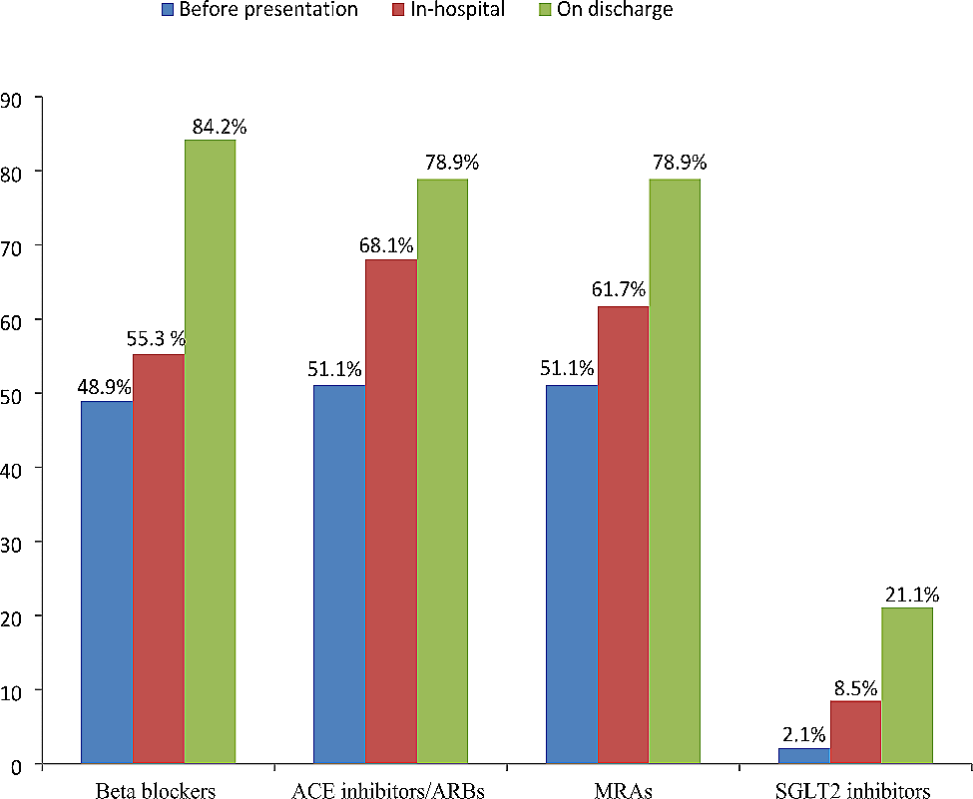
Prescription patterns of anti-remodeling therapies for patients with acute decompensated heart failure and reduced ejection fraction at Y12HMC (July 1, 2022 - July 1, 2023) MRAs, mineralocorticoid receptor antagonists; ACE, angiotensin converting enzyme; ARBs, angiotensin receptor blockers; SGLT2, sodium-glucose cotransporter 2

The most prescribed anti-remodeling medications for ADHF patients with LVEF ≤ 40% on discharge were beta blockers (84.2%), followed by ACE inhibitors/ARBs (78.9%), and MRAs (78.9%), and the least prescribed were SGLT2 inhibitors (21.1%) [Fig. 4].

### In-hospital outcomes

The median length of the in-hospital stay was 9 days (IQR: 6–14). From a total of 303 hospitalized patients with AHF, there were 26 deaths, making the in-hospital mortality rate 8.6%. The mortality rate was higher in the medical ICU (31.8%) compared to that of the medical wards (6.8%), and the majority of in-hospital deaths (61.5%) were due to cardiovascular causes, while the rest were due to non-cardiovascular causes. Most of the patients (89.9%) improved after their hospital stay and were discharged home, whereas 6.1% were referred to another hospital, and the rest (4%) were discharged without improvement, absconded, or left against medical advice.

### Factors associated with in-hospital mortality

The most important factors associated with in-hospital mortality of AHF in the bivariate logistic regression analysis were SBP < 115 mmHg [crude odds ratio (COR) = 5.83; 95% CI: 2.14, 15.91; *P* value = 0.001], DBP < 60mmHg (COR = 3.66; 95% CI: 1.33, 10.09; *P* value = 0.012), hypochloremia or Cl level < 96 mg/dL (COR = 3.95; 95% CI: 1.62, 9.62; *P* value = 0.002), AST > 40 U/L (COR = 3.31; 95% CI: 1.43, 7.65; *P* value = 0.005), ALT > 40 U/L (COR = 2.39; 95% CI: 1.01, 5.70; *P* value = 0.048] [Table 4].

In the multivariate logistic regression analysis, SBP <115 mmHg,  BUN > 20 mg/dL, Cl level < 96 mg/dL, and the presence of dyslipidemia were significantly associated with in-hospital mortality of AHF. The odds of in-hospital mortality were 6.28 times higher among AHF patients with SBP < 115 mmHg as compared with those with ≥ 115 mmHg (AOR = 6.28; 95% CI: 1.99, 19.78; *P* value = 0.002). The odds of in-hospital mortality were 4.88 times higher among AHF patients with a chloride level < 96 mg/dL as compared with those with ≥ 96 mg/dL (AOR = 4.88; 95% CI: 1.30, 18.33; *P* value = 0.019). The odds of in-hospital mortality were 5.48 times higher among AHF patients with a BUN level > 20 mg/dl as compared with those with a BUN value ≤ 20 mg/dl (AOR = 5.48; 95% CI: 1.47, 20.49; *P* value = 0.012). In addition, the odds of in-hospital mortality were 3.73 times higher in AHF patients with dyslipidemia as compared with those without dyslipidemia (AOR = 3.73, 95% CI: 1.15, 12.07, *P* value = 0.028). [Table 4].

**Table 4:** Bivariate and multivariate logistic regression analysis to identify predictors of in-hospital mortality in patients with acute heart failure at Y12HMC (July 1, 2022 - July 1, 2023)

Predictors	Death (No.)		Bivariate analysis		Multivariate analysis	
	Yes	No	COR	95% CI	AOR	95% CI
NYHA class						
IV	21	192	1.86	(0.68, 5.10)	1.03	(0.31, 3.49)
II/III	5	85	1		1	
SBP category						
<115	21	116	5.83	(2.14, 15.91)*	6.28	(1.99, 19.78)*
≥115	5	161	1		1	
DBP category						
< 60	6	21	3.66	(1.33, 10.09)*	2.08	(0.55, 7.81)
≥ 60	20	256	1		1	
BUN						
>20	11	74	2.94	(0.90, 9.63)	5.48	(1.47, 20.49)*
≤20	4	79	1		1	
Na^+^						
<135	12	89	1.62	(0.72, 3.65)	0.44	(0.13, 1.51)
≥ 135	14	168	1		1	
Cl ^–^						
<96	16	82	3.95	(1.62, 9.62)*	4.88	(1.30, 18.33)*
≥96	8	162	1		1	
AST						
>40	13	58	3.31	(1.43, 7.65)*	1.26	(0.38, 4.20)
≤40	12	177	1		1	
ALT						
>40	10	54	2.39	(1.01, 5.70)*	2.35	(0.61, 8.98)
≤40	14	181	1		1	
QRS prolongation						
Yes	5	30	3.00	(0.96, 9.39)	0.99	(0.20, 5.03)
No	10	180	1		1	
LVEF						
≤40	10	75	2.04	(0.81, 5.11)	1.94	(0.57, 6.59)
>40	10	153	1		1	
Dyslipidemia						
Yes	6	36	2.01	(0.76, 5.34)	3.73	(1.15, 12.07)*
No	20	241	1		1	

COR, crude odds ratio; AOR, adjusted odds ratio; NYHA, New York Heart Association; SBP, systolic blood pressure; DBP, diastolic blood pressure; BUN, blood urea nitrogen; Na, sodium; K, potassium; Cl, chloride; AST, aspartate transaminase; ALT, alanine transaminase; LVEF, left ventricular ejection fraction. *P value < 0.05

## Discussion

The total number of annual admissions due to acute heart failure (AHF) in this study was 311, which accounted for 13.2% of overall annual medical admissions (*N* = 2351). This falls in the range reported by the hospital prevalence studies done in Sub-Saharan Africa (SSA), where HF comprised 9.4–42.5% of all medical admissions [14]. Compared to the results of this study, advanced HF accounted for a higher percentage (18.2%) of annual medical admissions (*N* = 1165) in the study done at Saint Paul’s Hospital Millennium Medical College (SPHMMC) [15]. The reason for the higher percentage of AHF patients in the latter study may be due to the setting, which is characterized by a higher prevalence of advanced cardiovascular cases.

The mean age of patients in the current study was 56.7 (SD: 18.4) years, and 51.5% were females, showing similarity with the demographic characteristics of 1006 patients included in the THESUS-HF study, where the mean age was 52.3 years (SD: 18.3) and 50.8% were women [6]. In contrast, the Large-Scale Japanese Registry of ADHF revealed that the mean age was 78.0 ± 12.5 years; 68.9% of the patients were elderly (> 75 years) and 52.8% were males. This implies that AHF affects younger individuals in developing nations as compared to developed nations, but there are no such gender-based differences in its prevalence [16].

The commonest symptom and sign in the current study were dyspnea (98.7%) and peripheral edema (79%), respectively. Similar patterns of clinical findings, dyspnea (88.05%) and peripheral edema (80.5%), were reported as the commonest symptom and sign, respectively, from the study done at Gondar University Comprehensive Specialized Hospital (GUCSH) [8]. This underscores the continued importance of clinical findings in the diagnosis and management of HF in various healthcare settings. Cor pulmonale (22.8%) and HHD (17.8%) were the commonest etiologies of AHF in the current study, whereas the THESUS–HF study showed that HHD and dilated CMP were the leading causes of AHF [6] and the INTER-CHF revealed that the commonest causes of HF were IHD (20%) and HHD (35%) [7]. In contrast, IHD was the leading cause of AHF (27%) in the study from GUCSH [8], and RHD was the most common underlying cause for AHF in the studies done at SPHMMC (30%) and TASH (48.5%) [8, 10], where a lot of RHD cases are referred for interventional therapies. This underscores the impact of patient characteristics, referral systems, and healthcare infrastructure on the distribution of etiologies of AHF in different settings.

The current study revealed that pneumonia (35.3%) was the most common precipitating factor for AHF, followed by atrial fibrillation (16.8%) and drug discontinuation (14.5%). In line with the results of this study, pneumonia (47.5%) was the most frequent precipitating factor of AHF in a prospective observational study conducted on 165 patients at Tikur Anbessa Specialized Hospital [10]. In addition, pneumonia was the most frequent cause of HF exacerbation among the 1645 eligible Egyptians who were included in the ESC-HF-LT Registry [17]. Though pneumonia was found to be the leading precipitating factor for AHF in the current and several other studies, it could have been misdiagnosed on some occasions since it masquerades as pulmonary congestion in terms of clinical and/or radiological findings.

Among the electrolyte disorders, sodium abnormality has gotten strong attention in many of the previous studies done on AHF; for instance, a study done in tertiary care hospitals in Ethiopia reported hyponatremia as the commonest electrolyte disorder (43%) in patients with AHF [18]. However, none of the studies in Ethiopia have reported the prevalence of chloride disorder in AHF. On the contrary, the current study revealed that hypochloremia was the most common electrolyte abnormality (36.6%), which is higher than that reported in the PROTECT study (13%) [19]. Our study highlights the importance of considering chloride abnormality as one major electrolyte disturbance in patients with AHF. The majority (60.9%) of patients in the current study had HFpEF, and likewise, the study done at TASH showed a predominance (74.3%) of HFpEF [10]. However, the China HF Center Registry Study showed a lower percentage (43%) of patients with HFpEF [20]. The higher percentage of patients with HFpEF in our study corresponds to the leading etiologies (cor pulmonale, HHD, and IHD), which are not usually accompanied by a reduction in LVEF.

In the current study, furosemide (96%) and antibiotics (62.4%) were the most commonly prescribed medications during hospitalization. Similarly, furosemide was the most utilized drug during hospitalization (98.9%), and antibiotics were prescribed to 66.4% of patients in a study done on admitted AHF patients at tertiary care hospitals in Ethiopia [18]. The most commonly prescribed anti-remodeling agents on discharge were BBs (47.7%), and MRAs (42.8%), followed by ACE inhibitors/ARBs (38.6%), whereas in the study done at SPHMMC, the most prescribed anti-remodeling agents on discharge were spironolactone (71%), ACE inhibitors (38.9%) and BBs (27.9%) [8]. It seems that there was overzealous use of antibiotics, which could be due to overdiagnosis of pneumonia masquerading as lung congestion of AHF. On the contrary, the use of anti-remodeling agents looks suboptimal and the utilization of SGLT2 inhibitors was very low, which might be due to clinical inertia or inaccessibility of the medications.

The overall in-hospital rate (8.6%) and median length of hospital stay (9 days) in patients with AHF in the current study were lower as compared with other studies in Ethiopia, where the study done at GUCSH showed a mortality rate of 10.6% and a median length of stay of 17 days and the study at SPHMMC revealed a mortality rate of 24.4% and a median length of stay of 11 days [8, 9]. A higher in-hospital mortality rate was also seen Dessie Referral Hospital (22.9%) [11], Jimma University Medical Centre (21.3%) [12], and TASH (17.2%) [10]. However, a lower mortality rate (7.7%) was observed in the Large-Scale Japanese Registry of ADHF (16). The variations in mortality and length of hospital stay might be related to differences in patient characteristics, healthcare infrastructure, or quality of care.

In line with the results of the ADHF National Registry (ADHERE), the current study has found that high BUN values (> 20 mg/dL) and SBP < 115 mm Hg were significantly associated with in-hospital mortality of AHF [21]. Hypochloremia has been implicated as one possible cause of grave prognosis in patients with ADHF [22], and this has been replicated in the current study. Likewise, the analysis of the TOPCAT trial revealed that low chloride level was associated with an increased risk of cardiovascular death, and all-cause mortality [23]. The presence of dyslipidemia was associated with in-hospital mortality in the current study, and this was consistent with a study on Saudi patients with HF, where low levels of high-density lipoprotein cholesterol contributed to significant mortality risk [24]. Conversely, the Get With the Guidelines-HF registry showed that a lower total cholesterol level predicts a higher risk of in-hospital death in HF patients [25]. The findings of our study indicate the clinical relevance of the need for monitoring of renal function, avoiding lower SBP ranges, and addressing abnormal chloride levels and lipid profiles as part of comprehensive care for AHF patients to improve their in-hospital mortality. .

This study has some limitations. First, because of the retrospective data collection from the electronic medical record system, the accuracy of the recorded data may affect the results. Secondly, the cross-sectional nature of our study precludes determining a cause-and-effect relationship. Finally, the results may not be generalizable to ambulatory chronic HF patients, as this study included only hospitalized acute HF patients.

## Conclusions

This study revealed that the annual admission rate of acute heart failure (AHF) at the medical wards and ICU of Yekatit 12 Hospital Medical College, Addis Ababa, Ethiopia, was 13.2%. Importantly, the in-hospital mortality rate due to AHF was found to be lower in comparison to the previous studies conducted in various areas of the country. Factors such as systolic blood pressure (SBP) < 115 mmHg, blood urea nitrogen (BUN) > 20 mg/dL, chloride (Cl) level < 96 mg/dL, and the presence of dyslipidemia were significantly associated with in-hospital mortality for AHF patients. Hence, healthcare providers need to stratify AHF cases on admission based on their risk of in-hospital mortality and address those potential negative prognostic indicators. Besides, the respective stakeholders and experts should develop and implement strategies aimed at improving the standard of care for AHF patients and reducing in-hospital mortality further.

## Data Availability

All data generated or analyzed during this study are included in this manuscript.

## Abbreviations

ADHF: Acute decompensated heart failure
AF: Atrial fibrillation
AHF: Acute heart failure
CMP: Cardiomyopathy
COPD: Chronic obstructive pulmonary disease
COVID-19: Coronavirus disease 2019
DBP: Diastolic blood pressure
RHD: Rheumatic heart disease
DVHD: Degenerative Valvular Heart Disease
EF: Ejection fraction
HF: Heart Failure
HFmrEF: Heart failure with mildly reduced ejection fraction
HFpEF: Heart failure with preserved ejection fraction
HFrEF: Heart failure with reduced ejection fraction
HTN: Hypertension
IHD: Ischemic Heart Disease
ICU: Intensive care unit
LVEF: Left ventricular ejection fraction
NYHA: New York Heart Association
SBP: Systolic blood pressure
